# Carrier-Free Immobilization of α-Galactosidase as Nano-Biocatalysts for Synthesizing Prebiotic α-Galacto-Oligosaccharides

**DOI:** 10.3390/molecules26051248

**Published:** 2021-02-25

**Authors:** Yan Liu, Jingyi Yang, Ke Wang, Feiyu Duan, Lili Lu

**Affiliations:** School of Pharmacy, Tongji Medical College, Huazhong University of Science and Technology, 430030 Wuhan, China; lyan3356@163.com (Y.L.); M201978271@hust.edu.cn (J.Y.); D201881327@hust.edu.cn (K.W.); M201975462@hust.edu.cn (F.D.)

**Keywords:** α-galactosidase, immobilization, cross-linked enzyme aggregates, α-galacto-oligosaccharides, response surface methodology, batch synthesis

## Abstract

α-Galacto-oligosaccharides (α-GOSs) have great functions as prebiotics and therapeutics. This work established the method of batch synthesis of α-GOSs by immobilized α-galactosidase for the first time, laying a foundation for industrial applications in the future. The α-galactosidase from *Aspergillus niger* L63 was immobilized as cross-linked enzyme aggregates (CLEAs) nano-biocatalyst through enzyme precipitating and cross-linking steps without using carriers. Among the tested agents, the ammonium sulfate showed high precipitation efficacy and induced regular structures of α-galactosidase CLEAs (Aga-CLEAs) that had been analyzed by scanning electron microscopy and Fourier-transform infrared spectroscopy. Through optimization by response surface methodology, the ammonium sulfate-induced Aga-CLEAs achieved a high activity recovery of around 90% at 0.55 U/mL of enzymes and 36.43 mM glutaraldehyde with cross-linking for 1.71 h. Aga-CLEAs showed increased thermal stability and organic solvent tolerance. The storage ability was also improved since it maintained 74.5% activity after storing at 4 °C for three months, significantly higher than that of the free enzyme (21.6%). Moreover, Aga-CLEAs exhibited excellent reusability in the α-GOSs synthesis from galactose, retaining above 66% of enzyme activity after 10 batch reactions, with product yields all above 30%.

## 1. Introduction

Prebiotics are non-digestible food fibers that beneficially affect the host health by selectively increasing the growth and activity of gut microbes, especially *Bifidobacteria* and *Lactobacilli*, and also by their effects on system-wide metabolic and physiological readouts [1]. α-Galacto-oligosaccharides (α-GOSs) have attracted particular attention due to the prebiotic role as strong Bifidus growth factors in maintaining gut microflora balance. They significantly stimulate the growth of intestinal probiotics, resulting in a series of beneficial effects such as enhanced intestinal health, improved immune activities, alleviation of inflammatory response, reduction of cancer risk, and so on [2,3,4,5,6]. Recently, α-GOSs have been found with promise as therapeutics to antagonize cancer and infective diseases, exemplified by the stachyose-induced cancer apoptosis through the caspase-dependent mitochondrial pathway as well as the raffinose mediated inhibition of pathogen biofilm formation by attaching to an essential carbohydrate-binding protein [7,8]. Due to their importance, the access to α-GOSs in large quantity has drawn considerable interest. Unfortunately, the natural source extraction from plants is complex and uneconomical owing to low content, while the chemical synthesis requires tedious steps to control stereo- and regioselectivity of sugars. In contrast, the enzymes enable the one-step synthesis of target glycoside linkages with specificity in an environment-friendly manner [9].

**Figure A1:**
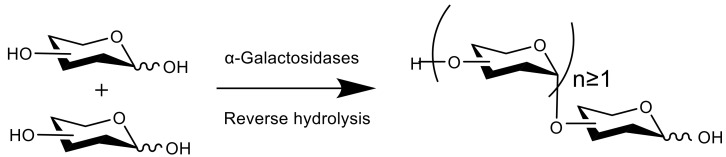
α-Galactosidases-catalyzed oligosaccharide synthesis by reverse hydrolysis. The monosaccharide galactose is used as glycosyl donor. The monosaccharide and newly produced oligosaccharides can be used as glycosyl acceptor.

α-Galactosidases (EC 3.2.1.22), an important class of glycosidases that naturally catalyze the hydrolysis of terminal galactose from oligosaccharides and glycoconjugates, have been investigated and applied in the synthesis of important α-GOSs or α-galactosides in vitro [10,11,12,13,14]. The transglycosylation reactions commonly catalyzed by the enzymes require melibiose or nitrophenyl-α-*D*-galactopyranoside as glycosyl donors, which are expensive for large-scale synthesis. Interestingly, α-galactosidases also have the ability to catalyze reverse hydrolysis by employing monosaccharide, galactose, as the glycosyl donor (Figure A1), which is significantly cheaper than the oligosaccharide or glycoside donors that are used for transglycosylation. Moreover, the hydrolysis of glycosyl donors existing in the transglycosylation process will not happen when utilizing the monosaccharide donor, making the product mixtures simple and easy to purify. The α-galactosidases from *Aspergillus niger* APC-9319, *Candida guilliermondii* H-404, and *Bifidobacterium bifidum* NCIMB41171 have been found to synthesize α-GOSs from galactose [15,16,17,18]. 

Notwithstanding the advantages of α-galactosidase-mediated synthesis, all the reported reactions were catalyzed by the free enzymes that have some limits in long-term operations for industrial applications. It is known that the free enzymes, which require unique three-dimensional structures for their activities, are sensitive to environmental conditions and easy to denature when exposed to certain conditions such as elevated temperatures or organic solvents [19]. Furthermore, the free enzymes are generally soluble in aqueous solutions and hard to recover from product mixtures for reuse. These problems can generally be overcome by immobilization of enzymes to solid supports or their self-assembly into insoluble particles, which strongly improves their properties such as enhanced storage and operational stability in changing environments, facile separation, and reusability in continuous or batch biocatalytic processes [20,21]. 

The traditional immobilization strategies comprise enzyme adsorption, encapsulation, chemical or enzymatic cross-linking to a carrier material. Alternatively, there is a recently developed strategy that affords carrier-free immobilized enzymes with high productivities and avoids the costs of the carrier. The enzyme proteins are firstly precipitated by salts, organic solvents, or nonionic polymers and then cross linked by glutaraldehyde to form insoluble biocatalysts designated as cross-linked enzyme aggregates (CLEAs), a class of nano-biocatalysts with sizes ranging from tens to hundreds of nanometers [19,20,21,22]. CLEAs of the α-galactosidase from maize have been used for oligosaccharide hydrolysis [23].

In this work, the synthesis of α-GOSs by the α-galactosidase immobilized in the CLEAs form was for the first time established. The carrier-free immobilization was performed by enzyme precipitating and cross-linking steps. Various precipitants were screened and the cross-linking conditions were optimized by response surface methodology (RSM), a statistical method with overall consideration of the interactive effects among various factors [24], leading to a high enzyme activity recovery after immobilization. The immobilized nano-biocatalyst was characterized and found to have higher thermal stability and organic solvent tolerance as well as longer service life than the free enzyme. It had been finally successfully applied in the batch synthesis of α-GOSs.

## 2. Results and Discussion

### 2.1. Screening of Precipitants for Inducing Aga-CLEAs Nano-Biocatalyst

**Figure 1:**
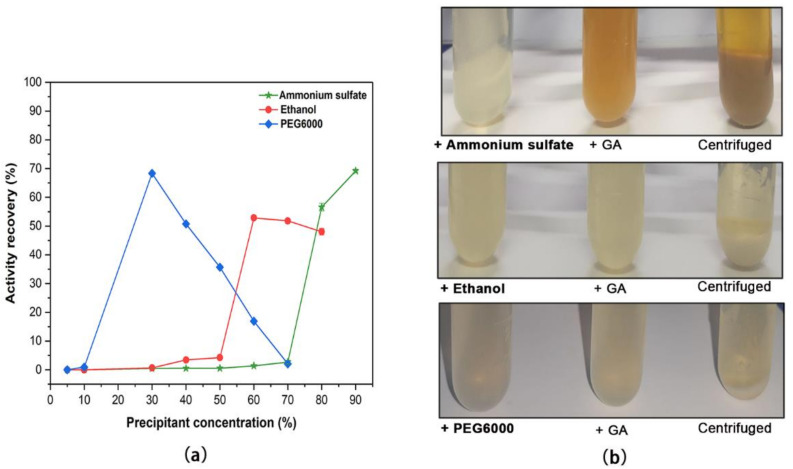
Preparation and structural analysis of α-galactosidase CLEAs. (**a**) The effects of different agents on precipitation of α-galactosidase. The tested precipitants were ammonium sulfate (30%–90% saturation at 4 °C), ethanol (5%–80%, *v/v*), and PEG6000 (5%–70%, *w/v*), respectively. (**b**) The process of CLEAs formation. The enzyme was precipitated, cross linked, and centrifuged to yield CLEAs that were distributed at the bottom of the tube. Ammonium sulfate, ethanol, and PEG6000 were used at 90%, 60%, and 30%, respectively. GA, glutaraldehyde.

*A. niger* L63 was cultured in the liquid medium and produced extracellular α-galactosidase with a maximal amount of 1029 U/L after six days. The culture was filtered, and the supernatant was collected and used as a crude enzyme for immobilization. The insoluble biocatalysts of Aga-CLEAs were prepared by precipitating the crude enzyme with various precipitant agents followed by glutaraldehyde-mediated cross-linking. The tested precipitants were a neutral salt (ammonium sulfate), a water-soluble organic solvent (ethanol), and a nonionic polymer (PEG6000). As shown in Figure 1a, all the tested precipitants could result in an above 50% activity recovery of α-galactosidase, with efficacy dependent on the concentrations. PEG6000 at 30% and ammonium sulfate at 90% precipitated about 70% α-galactosidase, while 60% of ethanol led to a maximal activity recovery of 55%. 

Next, the cross-linking conditions of Aga-CLEAs induced from different precipitants were preliminarily investigated and determined as follows: 50 mM glutaraldehyde at 4 °C for 2 h for ammonium sulfate-precipitated enzyme; 20 mM glutaraldehyde at 4 °C for 2 h for ethanol-precipitated enzyme; 15 mM glutaraldehyde at 4 °C for 3 h for PEG6000-precipitated enzyme. Figure 1b shows the process of Aga-CLEA preparation under the conditions mentioned above. The Aga-CLEAs prepared from the three precipitants were apparently observed at the bottom of the tube after centrifugation. The cross-linking solution of ammonium sulfate became yellow due to the chemical reactions with glutaraldehyde, but it did not affect the α-galactosidase activity. 

**Figure A2:**
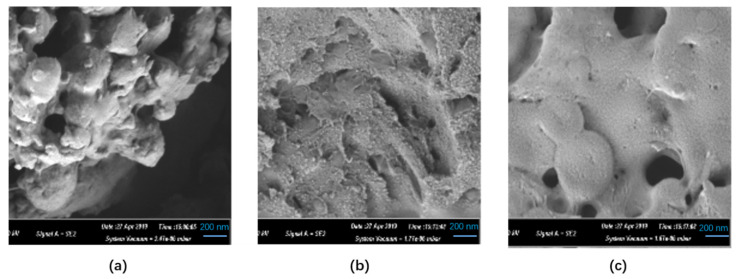
SEM analysis of CLEAs. (**a**) Enzyme + ammonium sulfate (90%) + glutaraldehyde (50 mM); (**b**) Enzyme + ethanol (60%) + glutaraldehyde (20 mM); (**c**) Enzyme + PEG6000 (30%) + glutaraldehyde (15 mM).

It was reported that the way the enzymes packed together in CLEAs had a critical impact on the aggregate activity. Two different structures of enzyme aggregates have been previously discovered: one is regular “balls’’ (Type 1) with better mass transfer and the other is less-defined structures with more hydrophilic residues on the surface (Type 2) [25,26]. To disclose the patterns of various Aga-CLEAs, the enzyme samples were subjected to SEM analysis (Figure A2). In comparison with the control sample that was loosely packed without cross linking, the ammonium sulfate-induced Aga-CLEAs were clustered into “balls” with approximately 250 nm in diameter (Figure A2-a), similar to Type 1 aggregate. In the case of PEG6000-induced Aga-CLEAs, only a few spherical structures were observed (Figure A2-c). As for ethanol-induced Aga-CLEAs, the entire structures seemed irregular with all enzyme molecules packed together (Figure A2-b), which would increase mass-transport limitations. Therefore, the ammonium sulfate was selected as the precipitant to induce Aga-CLEAs nano-biocatalyst in the following experiments considering the good precipitation efficacy as well as the regular CLEA pattern.

**Figure 2:**
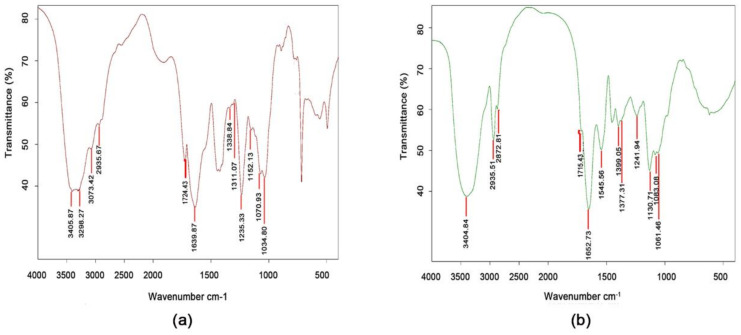
FTIR spectroscopy analysis. (**a**) The control without cross-linking; (**b**) The ammonium sulfate-induced CLEAs.

The structures of ammonium sulfate-induced Aga-CLEAs were further analyzed by FTIR spectroscopy, using the free enzyme aggregates without cross-linking as the control (Figure 2). It should be noted that the FTIR spectra verified the cross-linking reactions in the ammonium sulfate-induced Aga-CLEAs. The N-H signals changed from doublet (3405.87 cm^−1^, 3298.27 cm^−1^) to singlet (3404.84 cm^−1^) after enzyme immobilization, indicating the depletion of a primary amine in the cross-linking process. Also, the C=O band (1600–1850 cm^−1^) signals changed significantly, with an increased C=O signal from the amide group and a reduction in C=O intensity from the carboxyl acid group, which was mainly due to the participation of the free carboxylic acid groups in the formation of amide groups during cross-linking process. In addition, Aga-CLEAs showed an intense C-N signal at 1545.56 cm^−1^ that was absent in the free enzyme. This directly proved the formation of the C-N bond during the cross-linking process, where the free amino groups on the surface of the enzyme protein had cross linked with the aldehyde group of glutaraldehyde to form Schiff base [27,28]. 

### 2.2. Optimization of the Cross-Linking Conditions for Aga-CLEAs by RSM

The preparation of ammonium sulfate-induced Aga-CLEAs was further optimized by the statistical RSM, which had an overall consideration of the interactive effects among various factors in the cross-linking process. The concentrations of glutaraldehyde were considered important to obtain highly active Aga-CLEAs since lower concentrations might lead to an incomplete cross-linking enzyme with low activity recovery, while higher concentrations would either inactivate the enzyme or result in tightly packed aggregates with increased mass-transfer resistance [29,30]. The cross-linking time was another crucial factor affecting the activity recovery, considering that too long of a processing time would lead to excessive cross-linking with an altered configuration of CLEAs [31]. The enzyme amount was related to the particle size of the CLEAs and also required to optimize [32]. Consequently, the factors of glutaraldehyde concentration (X_1_), cross-linking time (X_2_), and enzyme amount (X_3_) were investigated by RSM in order to achieve maximal activity recovery.

Table 1 shows the experimental matrices and results of the central composite design (CCD). By multiple regression analysis of the experimental data, the second-order polynomial equation was generated to explain the effect of the independent variables on the activity recovery as following:

Y = −685.49 + 24.30X_1_ + 2.23X_2_ + 1103.08X_3_ + 0.30X_1_X_2_ − 18.64X_1_X_3_ + 242.30X_2_X_3_ − 0.20X_1_^2^ − 42.85X_2_^2^ − 668.36X_3_^2^ (1)

Analysis of variance (ANOVA) was performed to verify the fit and adequacy of the established mathematical model (Table A1). The model had a small Prob > F value (<0.0001), indicating a good correlation with the experimental results. It was known that the accuracy of fit of the polynomial model equation was expressed by the coefficient of determination (R^2^), of which the closer the value is to 1.00, the better the model predicts the response. In Table A1, the value of R^2^ was 0.9481, indicating that 94.81% of the variability in the response can be explained by the model. The adjusted determination coefficient (Adj R^2^) was also at a very high value of 0.9015, which advocated the high significance of the model. The F value (0.42) of the lack of fit was insignificant, supporting the reliability of the model. Meanwhile, a low value of the coefficient of variation (7.26%) indicated good reliability of the experimental data for the model. All these analyses confirmed that the response Equation (1) provided a suitable model for the CCD experiments. Therefore, the investigation of the response trends using the model was reasonable. 

**Figure 3:**
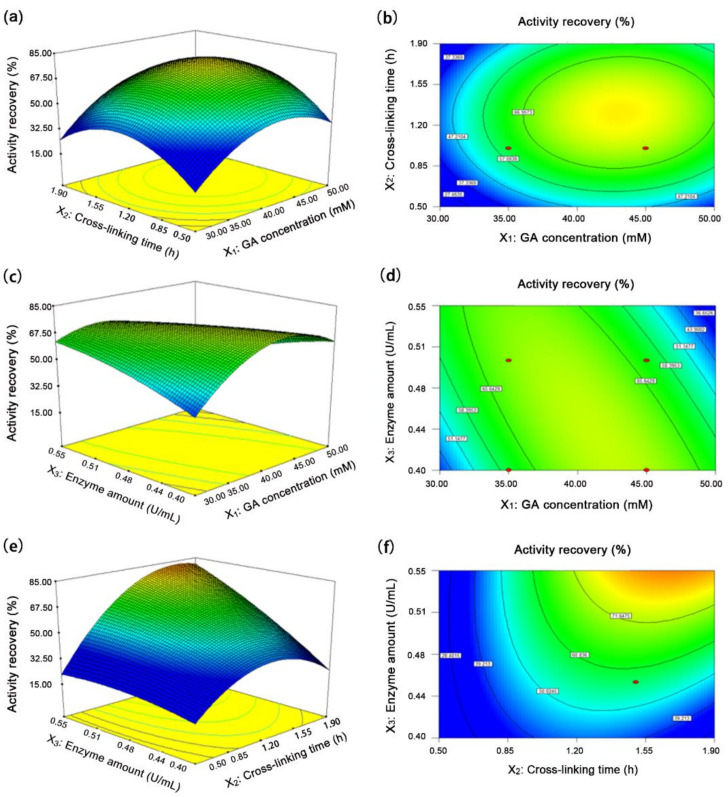
Three-dimensional (left) and two-dimensional (right) response surface plots of the interaction between different variables on activity recovery. (**a**,**b**) the effects of glutaraldehyde concentration and cross-linking time on activity recovery; (**c**,**d**) the effects of glutaraldehyde concentration and enzyme amount on activity recovery; (**e**,**f**) the effects of cross-linking time and enzyme amount on activity recovery. GA, glutaraldehyde.

The optimal level of each factor and the effect of their interaction on activity recovery were further explored by constructing three-dimensional response surface and two-dimensional contour plots based on the regression model (Figure 3). The maximal activity recovery of Aga-CLEAs was estimated to be 90.70% under the conditions of 36.43 mM glutaraldehyde, 0.55 U/mL enzyme, and 1.71 h cross-linking as analyzed by Design-Expert 8.0. To verify the adequacy of the model equation for the predicted maximal activity recovery, three parallel experiments were carried out under the theoretically optimal conditions. The average activity recovery turned out to be 90.86%, closely consistent with the predicted value, which proved the good correlation between the theoretical and experimental results and justified the validity of the response model.

### 2.3. Biochemical Properties of Aga-CLEAs Nano-Biocatalyst

The Michaelis–Menten constants for the free and immobilized enzymes were calculated. The *K*_m_ value of Aga-CLEAs (0.37 ± 0.05 mM) for *p*NP-α-Gal was slightly increased in comparison with that of the free enzyme (0.30 ± 0.02 mM), which might be possibly due to the mass transfer resistance between the aggregated enzyme and the substrate [21]. A similar phenomenon had been found in the CLEAs of glucoamylase, which exhibited a higher *K*_m_ value of 1.16 ± 0.089 mg/mL toward maltodextrin than the free enzyme (0.63 ± 0.024 mg/mL) [33]. The *V*_max_ value of Aga-CLEAs was 7.53 ± 0.15 µmol/min, slightly higher than that of the free enzyme (5.81 ± 0.08 µmol/min), suggesting the immobilization manipulation did not impede the reaction rate.

**Figure 4:**
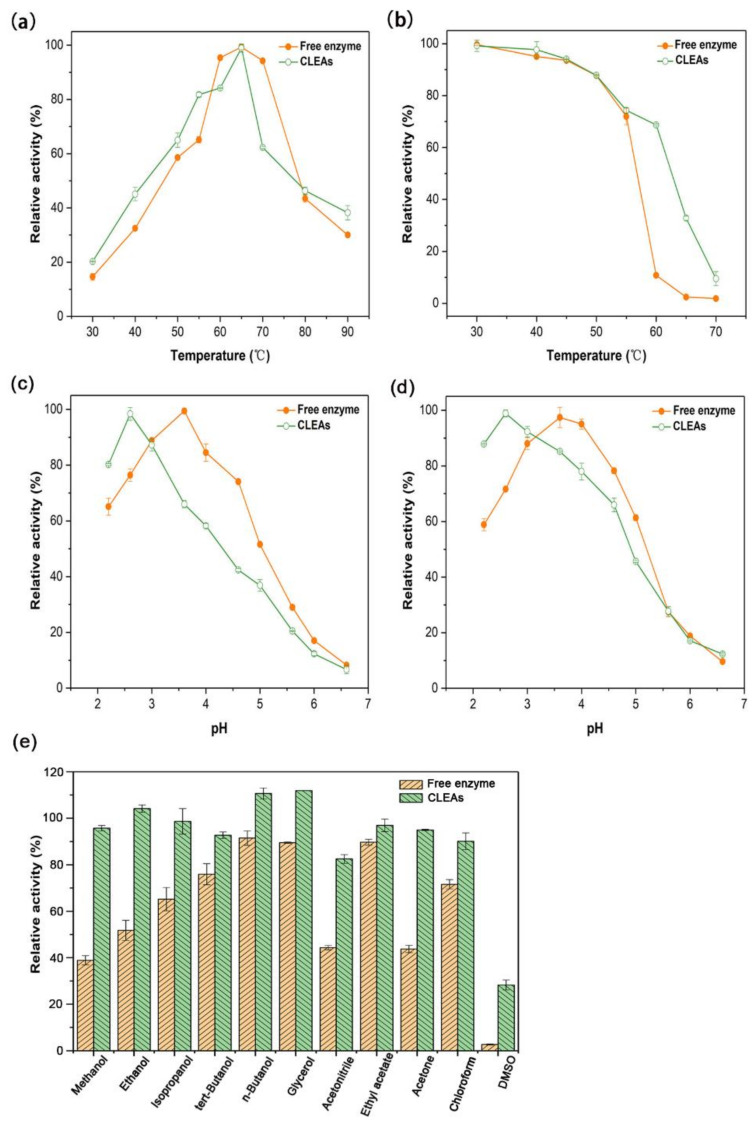
The effects of temperature, pH, and organic solvents on activities and/or stabilities of the free enzyme and Aga-CLEAs. (**a**,**b**) the effects of temperature on the activity and stability, respectively; (**c**,**d**) the effects of pH on the activity and stability, respectively; (**e**) the stability of the free enzyme and CLEAs in the presence of different organic solvents at 10% (*v/v*) concentration. Data points are the average of triplicate measurements, and error bars represent standard deviation.

The free and immobilized enzymes were both highly active at 60 °C and stable below 55 °C, remaining above 80% residual activity after 2 h incubation (Figure 4a,b). When the temperature was continuously elevated, the thermal stability of Aga-CLEAs was significantly higher than the free enzyme. The immobilized enzyme maintained about 70% residual activity at 60 °C, whereas the free enzyme was only 10% of their initial activity at the same temperature (Figure 4b). The enhanced thermo-stability by immobilization had been observed in several studies of CLEAs. One example was that the CLEAs of poly-3-hydroxybutyrate (PHB) depolymerase from *Streptomyces exfoliatus* maintained 50% of its initial activity after incubation at 70 °C for 40 min, while the soluble counterpart was completely deactivated under identical processing conditions [34]. Another example was that the combined CLEAs of papain (EC 3.4.22.2) and neutrase (EC 3.4.24.4) retained 70.55% activity after the treatment at 50 °C for 50 min, whereas the free enzymes presented only 3.78% activity [35]. Additionally, the CLEAs of peroxidase from *Brassica rapa* kept approximately 35% activity after incubation at 70 °C for 15 min, whereas the free enzyme was totally inactivated after the same treatment [36]. The improvement of thermal stability might be related to the more rigid structures as cross-linking aggregates when compared to the free enzyme [37].

The optimal pH values of the free and immobilized enzymes were 3.6 and 2.6, respectively (Figure 4c). The pH preference of Aga-CLEAs shifted to an acidic direction after immobilization. Similarly, Aga-CLEAs showed better stability in the acidic environment than the free enzyme (Figure 4d). The low pH value (2.6) at which the Aga-CLEAs were active and stable would inhibit microbe growth and help prevent microbial contaminations, making the immobilized enzyme especially suitable for long-term operations.

As shown in Figure 4e, Aga-CLEAs exhibited obviously higher stabilities toward all the tested organic solvents than the free enzyme. It kept above 80% activity in the presence of methanol, acetonitrile, and acetone, whereas the free enzyme remained only less than 50% activity under the same conditions. Even in the presence of DMSO that could fully inactivate the free enzyme, the immobilized enzyme could still retain above 20% activity. The enhanced tolerance of the immobilized enzyme toward organic solvents has also been found in PHB depolymerase CLEAs [34]. It seemed that cross-linking as aggregates protected the enzyme proteins from exposure to the organic solvent and thus improve the tolerance and stability. This property of CLEAs was beneficial for applications in aqueous two-phase or multiphase systems with advantages in avoiding microbial infection and reducing product hydrolysis. 

### 2.4. Storage Stability of Aga-CLEAs Nano-Biocatalyst

The storage stability of Aga-CLEAs was investigated at 4 °C for three months. During the initial 40 days, both free and immobilized enzymes could retain above 80% activity (Table 2). After 60 days, however, the activity of free enzymes dropped sharply to 65.2%. In contrast, the change of Aga-CLEA activity was slight, keeping above 87.4% activity during the first two months. When continuously extended to three months, Aga-CLEAs could still maintain a high residual activity of 74.5%, whereas the activity of the free enzyme significantly reduced to 21.6%. It was obvious that the immobilized enzyme displayed higher activity than the free enzyme when the storage time was prolonged. The long-term storage stability of immobilized enzymes was significant for commercial applications since it would extend the service life of enzymes and cut down the cost for industrial productions. 

### 2.5. Batch Synthesis of α-GOSs by Recycling Aga-CLEAs Nano-Biocatalyst

**Figure 5:**
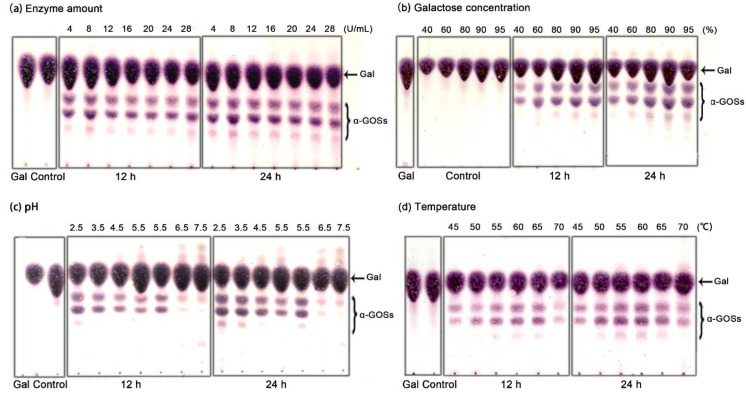
The effect of reaction conditions on α-GOSs synthesis by Aga-CLEAs. (**a**) The effect of enzyme amount (4 to 28 U/mL); (**b**) The effect of galactose concentration (40 to 95%, *w/v*); (**c**) The effect of reaction pH (2.5 to 7.5); (**d**) The effect of reaction temperature (45 to 70 °C). The control reactions were performed by incubation of inactivated enzyme with galactose for 24 h. Gal, galactose.

**Figure 6:**
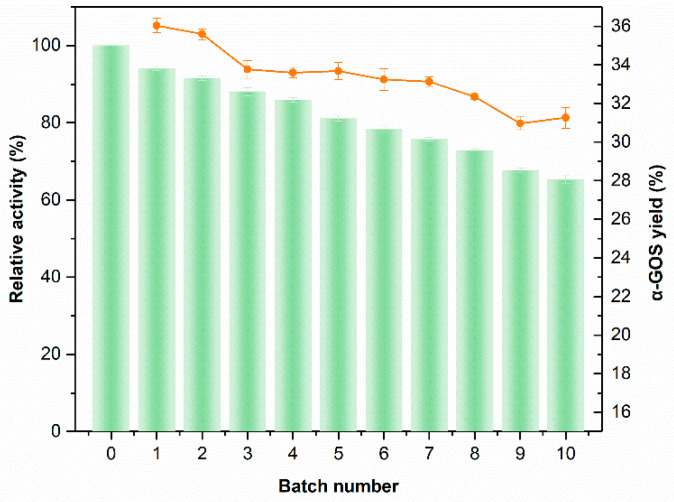
Batch synthesis of α-GOSs by recycling Aga-CLEAs. Column and line charts represent residual enzyme activity and oligosaccharide yield, respectively. Data represent the means of three experiments and error bars represent standard deviation.

The quality of the immobilized nano-biocatalyst was evaluated by recycling it in the batch synthesis of α-GOSs. The reaction conditions including the amount of Aga-CLEAs, the concentration of galactose, the pH of the buffer, the reaction temperature, and time on the production of α-GOSs were firstly investigated (Figure 5). The α-GOSs products could reach a high yield of about 36% by incubation of 8 U/mL Aga-CLEAs with 95% galactose in 50 mM citrate buffer (pH 2.5) at 60 °C for 19 h. The subsequent reactions of batch synthesis were performed under the same optimal conditions. As shown in Figure 6, the residual activity of Aga-CLEAs could maintain above 66% of initial activity after 10 batch reactions, with α-GOSs yields all keeping above 30%. The good stability and reusability of Aga-CLEAs would significantly reduce the operation cost, suggesting a great promise in industrial applications.

## 3. Materials and Methods

### 3.1. Materials 

*p*-Nitrophenyl α-*D*-galactopyranoside (*p*NP-α-Gal) was provided by Macklin (Shanghai, China). Silica gel 60 F254 plates were obtained from Merck (Darmstadt, Germany). Potatoes were purchased from Zhongbai Market (Wuhan, China). All other chemicals and reagents were of analytical grade such as *D*-galactose, 25% (*w/v*) glutaraldehyde in water, and organic solvents.

### 3.2. Production of α-Galactosidase

The strain of *A. niger* L63 was inoculated on potatoes dextrose agar (PDA) medium containing 20 g/L glucose and 15 g/L agar in the potato infusion that was prepared by boiling 200 g sliced potatoes in 1 L water for 30 min, followed by filtration through cheesecloth. After growth at 28 °C for three days, the spores of *A. niger* L63 were inoculated into the liquid medium comprising 10 g/L glucose, 10 g/L peptone, and 5 g/L yeast extract, with shaking at 200 rpm/min. After cultivation for two days at 28 °C, the cells of *A. niger* L63 were transferred into the same fresh medium in a ratio of 3% and incubated at 28 °C for six days. Then the culture broth was filtered and the supernatant was used as the crude enzyme for α-galactosidase.

### 3.3. Enzyme Activity Assay

The α-galactosidase activity was measured by adding 30 μL of enzyme solution or CLEAs suspension to 150 μL of 2 mM *p*NP-α-Gal in 50 mM acetate buffer (pH 5.5). The reaction was incubated in the metal bath with shaking at 400 rpm at 37 °C for 10 min and terminated by adding 1.05 mL of 0.2 M sodium borate (pH 10.5). The release of *p*-nitrophenol from *p*NP-α-Gal was detected at 400 nm in a spectrophotometer. One unit of enzyme activity (U) was defined as the amount of the enzyme required to release 1 μmol of *p*-nitrophenol per minute under the assay conditions. 

### 3.4. Preparation of α-Galactosidase CLEAs

The preparation of α-galactosidase CLEAs (Aga-CLEAs) consisted of two steps: initial precipitation and subsequent cross-linking. The precipitation step was performed by respectively mixing 200 µL of crude enzyme with 300 µL of three different precipitants, stirring at 4 °C for 1 h. The final concentrations of the precipitants, ammonium sulfate, ethanol, and PEG6000, were investigated at 30%–90% (saturation at 4 °C), 5%–80% (*v/v*), and 5%–70% (m/v), respectively. The α-galactosidase aggregates were obtained by centrifugation at 9391 rcf for 5 min and suspended in 50 mM sodium acetate—acetic acid buffer (pH 5.5). The enzyme activity recovery (%) was calculated based on the ratio of the remaining α-galactosidase activity of the precipitated enzyme aggregates to the total α-galactosidase activity of the initial crude enzyme before precipitation. 

The enzyme aggregates prepared from the above precipitants at the optimal concentrations were further cross linked by glutaraldehyde at concentrations ranging from 5 to 80 mM at 4 °C for 2 h. The cross-linking time and temperature were preliminarily investigated from 0.5 to 5 h and at 4 to 40 °C, respectively. The resulting cross-linked suspension was centrifuged at 9391 rcf for 5 min. The immobilized enzyme was collected and washed with 50 mM acetate buffer (pH 5.5) until no α-galactosidase activity was detected in the supernatant. The enzyme activity recovery (%) was calculated based on the ratio of the remaining α-galactosidase activity of Aga-CLEAs to the total α-galactosidase activity of the initial crude enzyme before cross-linking. 

### 3.5. Structural Analysis of Aga-CLEAs

For scanning electron microscopy (SEM) analysis, the enzyme samples were concentrated to powder by vacuum freeze-drying. The resulting dry samples were mounted on monocrystalline silicon, coated with gold on the surface, and detected by a ZEISS GeminiSEM field emission scanning electron microscope (Germany). 

For Fourier-transform infrared (FTIR) spectroscopy analysis, the dry enzyme samples as prepared above were mixed with KBr and pressed into a transparent sheet that was subsequently detected by the Bruker VERTEX 70/70v FTIR spectrometer (Germany) with spectra recorded in the range of 400 to 4000 cm^−1^. 

### 3.6. Optimization of Cross-Linking Conditions for Aga-CLEAs by Response Surface Methodology

The cross-linking conditions for the ammonium sulfate-induced Aga-CLEAs were optimized by the RSM that can investigate the interactions between the cross-linking factors and identify the optimal value of each factor to achieve the maximal activity recovery. The central composite design was employed to analyze the interaction among the significant factors including enzyme amount, cross-linking time, and glutaraldehyde concentration. The factors were tested at five levels (−1, +1, 0, −α, +α) and the experiments were arranged by the Design Expert software 8.0 (Table 3). The second-order polynomial coefficient was also calculated by the same software, and the role of each variable, their interactions, and the value of predicted response were explained through the following quadratic equation:

Y = β_0_ + Σ β_i_X_i_ + Σ β_ij_X_i_X_j_ + Σβ_ii_X_i_^2^ (2)

where *Y* is the predicted response (activity recovery); *X_i_* and *X_j_* represent the independent variables; *β*_0_ is a constant term; *β_i_* is the regression coefficient of linear terms; *β_ii_* is the quadratic regression coefficient; *β_ij_* represents interactive regression coefficient. 

### 3.7. Characterization of Aga-CLEAs Nano-Biocatalyst

Kinetic analyses of the free and immobilized enzymes were performed by incubation of the enzymes with *p*NP-α-Gal at different concentrations (0.3 to 2.2 mM) in 50 mM acetate buffer (pH 5.5). The reactions were incubated at 37 °C for 10 min and stopped by heating at 100 °C for 10 min. The released *p*-nitrophenol was detected at 400 nm by spectrophotometer. The *K*_m_ and *V*_max_ values of the enzymes were calculated by GraphPad Prism 6 software. 

The optimal temperature for the free and immobilized enzymes was determined by measuring the enzyme activity at temperatures ranging from 30 to 90 °C. The thermal stability of the enzymes was investigated by analysis of the residual activity after incubation at 30 to 70 °C for 2 h. The optimal pH for the free and immobilized enzymes was determined by testing the enzyme activity in the pH values ranging from 2.2 to 6.6 at 37 °C. The pH stability of the enzymes was determined by measuring the residual activity after incubation in the same pH range at 4 °C for 12 h. 

The stability of the free and immobilized enzymes in the organic solvents was studied by adding 10% (*v/v*) methanol, ethanol, isopropanol, *tert*-butanol, *n*-butanol, glycerol, acetonitrile, ethyl acetate, acetone, chloroform, or dimethyl sulfoxide (DSMO) in the enzyme assay mixture. The relative activity (%) was determined as the ratio of detected enzyme activity to the control reaction without organic solvents.

### 3.8. Storage Stability of Aga-CLEAs Nano-Biocatalyst

The storage stability of the free and immobilized enzymes was assayed by detecting enzyme activity of the enzyme samples that had been stored at 4 °C for three months. Aliquots were taken at 10, 20, 40, 60, 80, and 90 days for detecting the enzyme activities, respectively. The residual activity (%) was determined as the ratio of the remaining enzyme activity to the initial total enzyme activity before storage. 

### 3.9. Batch Synthesis of α-GOSs by Recycling Aga-CLEAs Nano-Biocatalyst

The α-GOSs synthesis was performed by incubation of the immobilized enzyme with galactose in the metal bath with shaking at 400 rpm. The impacts of reaction conditions on the production of α-GOSs were investigated in detail. The effects of the Aga-CLEAs amounts were tested at different concentrations ranging from 4 to 28 U/mL in the presence of 80% (*w/v*) galactose in 50 mM citrate buffer (pH 5.5) at 65 °C for 12 h and 24 h. The influences of the galactose concentrations were tested at 40%–95% (*w/v*) in a pH 5.5 buffer. A high concentration of galactose (96%, *w/v*) was prepared by autoclaving at 110 °C for 10 min and then adjusted to various concentrations by the addition of buffers. The effects of pH values were investigated at pH 2.5 to 7.5 by using 95% galactose as substrates. The influences of temperature were assayed by incubating the enzyme with 95% galactose (pH 2.5) at 45 to 70 °C, respectively. To study the effects of reaction time, assays were performed in enzyme reaction mixtures (pH 2.5) containing 95% galactose, which were incubated at 60 °C with aliquots serially analyzed within 24 h.

The batch synthesis of α-GOSs was carried out under the reaction conditions as optimized above. The initial batch reaction was started by incubating 8 U/mL of Aga-CLEAs with 95% galactose in 50 mM citrate buffer (pH 2.5) at 60 °C for 19 h with shaking. After one cycle of operation, the immobilized enzyme was separated from the reaction mixture by centrifugation at 10,000 rpm for 5 min, washed twice with 50 mM citrate buffer (pH 2.5), and resuspended in fresh 95% galactose in 50 mM citrate buffer (pH 2.5) for the next batch of reactions under the same conditions as the first batch. The reusability of the immobilized enzyme was assessed by comparing the residual enzyme activity after batch reactions with the initial α-galactosidase activity of Aga-CLEAs, combined with analyzing α-GOSs products. 

Oligosaccharide products were detected by thin-layer chromatography (TLC), in which the samples were loaded on the Silica gel 60 F254 plates and developed by a mixture solvent of n-butanol: ethanol: water (5:3:2, *v/v/v*). The TLC plates were sprayed with 0.5% (*w/v*) 3,5-dihydroxytoluene in 20% (*v/v*) sulfuric acid and the sugars on the plates would appear colors after heating at 120 °C for 5 min. The oligosaccharides were quantified by the software ImageJ v1.28 (http://rsb.info.nih.gov/ij/, accessed on 23 February 2021). The α-GOSs yield was defined as the mass ratio of oligosaccharide products to the total saccharides.

## 4. Conclusions

The method of α-GOSs synthesis by the α-galactosidase immobilized in the CLEAs form was developed for the first time. Ammonium sulfate was screened to exhibit high precipitation efficacy and induce regular structures of Aga-CLEAs nano-biocatalyst. Optimization of the cross-linking conditions by RSM resulted in a high enzyme activity recovery. The immobilized nano-biocatalyst showed excellent thermal stability and organic solvent tolerance along with long service life. It could be recycled in α-GOSs synthesis for at least 10 batches, with good enzyme activity and product yield. This low-cost production would lay a foundation for applications in large-scale α-GOSs synthesis. Continuous flow systems [38,39] will be tested to further extend the service life of Aga-CLEAs in future work.

## Figures and Tables

**Table 1:** Experimental design and results of the central composite design.

Run	Actual Level of Variables			
	Glutaraldehyde Concentration (mM)	Cross-Linking Time (h)	Enzyme Amount (U)	Activity Recovery (%)
1	45	1.00	0.50	62.32
2	35	1.00	0.50	72.17
3	40	1.50	0.45	85.80
4	30	1.50	0.45	54.67
5	45	1.00	0.40	70.88
6	40	1.50	0.35	63.45
7	40	1.50	0.45	79.71
8	45	2.00	0.50	67.86
9	35	2.00	0.50	74.92
10	40	1.50	0.45	73.30
11	35	2.00	0.40	40.61
12	40	0.50	0.45	40.83
13	50	1.50	0.45	67.86
14	35	1.00	0.40	62.32
15	40	1.50	0.45	89.41
16	40	1.50	0.45	79.55
17	45	2.00	0.40	52.42
18	40	2.50	0.45	35.98
19	40	1.50	0.55	85.69
20	40	1.50	0.45	77.88

**Table 2:** Storage stability of Aga-CLEAs at 4 °C.

Storage Time (days)	Residual Activity (%)	
	Free Enzyme	CLEAs
0	100 ± 0.4	100 ± 0.3
10	94.3 ± 0.7	98.9 ± 1.1
20	90.6 ± 1.5	97.5 ± 0.8
40	83.4 ± 2.1	94.2 ± 1.7
60	65.2 ± 1.4	87.4 ± 2.3
80	33.7 ± 0.9	79.8 ± 3.1
90	21.6 ± 3.4	74.5 ± 2.5

**Table 3:** Levels of the variables tested in the central composite design.

Variables	Symbol	Unit	Coded Levels				
			−α	−1	0	+1	+α
**Glutaraldehyde Concentration**	X_1_	mM	0.35	0.40	0.45	0.50	0.55
**Cross-Linking Time**	X_2_	h	0.5	1.0	1.5	2.0	2.5
**Enzyme Amount**	X_3_	U	30	35	40	45	50

## Sample Availability

Samples of the compounds are available from the authors.

## Appendix A

**Table A1:** ANOVA for response surface quadratic model ^a^.

Source	Sum of Square	Df	Mean Square	F Value	*p*-Value Prob ˃ F	
Model	4316.99	9	479.67	20.33	<0.0001	Significant
X_1_	686.74	1	686.74	29.09	0.0003	
X_2_	0.064	1	0.064	2.692 × 10^−3^	0.9596	
X_3_	137.81	1	137.81	5.84	0.0363	
X_1_ X_2_	4.56	1	4.56	0.19	0.6696	
X_1_ X_3_	173.72	1	173.72	7.36	0.0218	
X_2_ X_3_	293.55	1	293.55	12.44	0.0055	
X_1_^2^	627.86	1	627.86	26.60	0.0004	
X_2_^2^	2885.15	1	2885.15	122.23	<0.0001	
X_3_^2^	70.20	1	70.20	2.97	0.1153	
Residual	236.05	10	23.60			
Lack of fit	69.51	5	13.90	0.42	0.8202	not significant
Pure error	166.54	5	33.31			
Cor total	4552.34	19				

^a^ R^2^ = 0. 9481; AdjR^2^ = 0. 9015; Pred R^2^ = 0.8206; C.V. % = 7.26.
